# From 5F hematopoietic progenitors to osteoclasts: a scalable human model of osteoclastogenesis

**DOI:** 10.3389/fcell.2026.1773507

**Published:** 2026-06-08

**Authors:** Sarubala Malayaperumal, Sintra Stewart, Rachel Wellington, Sergei Doulatov, Elizabeth Leber, Marta Scatena, Alexander Blümke, Cecilia M. Giachelli

**Affiliations:** 1 Department of Bioengineering, School of Medicine, University of Washington, Seattle, WA, United States; 2 Division of Hematology, School of Medicine, University of Washington, Seattle, WA, United States; 3 Molecular and Cellular Biology Program, School of Medicine, University of Washington, Seattle, WA, United States; 4 Translational Science and Therapeutics Division, Fred Hutchinson Cancer Center, Seattle, WA, United States; 5 Department of Orthopedics and Trauma Surgery, Medical Faculty Mannheim, Heidelberg University, Mannheim, Germany

**Keywords:** 5F hematopoietic progenitors, bone resorption, engineered hematopoietic cells, hematopoietic differentiation, human induced pluripotent stem cells, osteoclast differentiation, osteoclastogenesis, osteoclasts

## Abstract

**Introduction:**

Human osteoclast models are essential for studying bone physiology and skeletal disease, yet existing systems are constrained by donor variability and the limited expansion capacity of hematopoietic progenitors.

**Methods:**

Conditionally expandable 5F hematopoietic stem and progenitor cells generated by transcription factor reprogramming with HOXA9, ERG, RORA, SOX4, and MYB were differentiated into osteoclasts. Cells were driven through an intermediate monocyte stage using IL three and M CSF, followed by RANKL stimulation to induce osteoclastogenesis.

**Results:**

Differentiated cells formed large, multinucleated, TRAP positive osteoclasts displaying F actin ring structures, cathepsin K expression, and strong bone resorptive activity. Gene expression analysis demonstrated marked upregulation of key osteoclast markers including NFATC1, MMP9, CTSK, and CA2.

**Discussion:**

By enabling conditional self-renewal at a downstream hematopoietic stage, the 5F platform overcomes key limitations of donor dependent and iPSC derived osteoclast models. This scalable and cytokine driven system provides a reproducible and standardized human osteoclast model suitable for studies of osteoclast biology, bone remodeling, and biomaterial interactions.

## Introduction

Osteoclasts (OCs) are specialized bone-resorbing cells that degrade the inorganic matrix and extracellular proteins by secreting acid and lytic enzymes. Originating from the monocyte lineage, these cells develop into large, multinucleated OCs (Boyle et al., 2003; Ansari et al., 2021; Vuoti et al., 2023). OCs have garnered increasing attention in bioengineering, regenerative medicine, and therapeutics, given their critical roles in bone remodeling, in evaluating how biomaterials interact with bone tissue, and potential therapeutic applications in conditions such as heterotopic ossification (Rementer et al., 2013; 2024; Jin et al., 2021). Investigating these roles and developing relevant therapeutic models begins with *in vitro* studies, which provide a controlled environment to assess drug effects and cellular behavior before progressing to *in vivo* studies and clinical translation. In this context, human-derived OCs are of particular importance, as they provide clinically relevant models for understanding bone physiology and for developing therapeutic approaches. However, since isolation of OCs directly from bone remains challenging, a variety of differentiation protocols have been established using alternative sources. Among cell lines, RAW264.7 remains the most widely used murine cell line (Hsu et al., 1999; Collin-Osdoby and Osdoby, 2012), while a few studies have employed human cell lines such as THP-1 (Li et al., 2017) and U937 (Arteaga and Guerrero, 2023; Ricchiuto et al., 2023). Despite their convenience, significant limitations remain. Murine RAW264.7 cells differ from human OCs in key signaling pathways, limiting their translational relevance (Rementer et al., 2013; Ng et al., 2018). Although human leukemia-derived lines such as THP-1 and U937 are easy to culture, their malignant origin raises concerns about physiological fidelity. As alternatives, primary cells such as peripheral blood mononuclear cells (PBMCs) or cells derived from bone marrow and cord blood have been extensively explored as a source for generating OCs (Flanagan and Massey, 2025; Cody et al., 2011; Abdallah et al., 2018; Madel et al., 2018; Rucci et al., 2019). Nevertheless, these approaches possess inherent drawbacks, such as limited availability of donor material and batch-to-batch variability. Such variability arises from donor-dependent factors such as differences in precursor frequency, adhesion efficiency and source-related differences, all of which complicate direct comparison of results (Xu et al., 2023). Our group has previously demonstrated OC differentiation from induced pluripotent stem cells (iPSCs) derived from fibroblasts and PBMCs, comparing the efficacy of different protocols (Blümke et al., 2023). We showed that iPSCs differentiated via the embryoid body (EB) method gave rise to large, multinucleated cells in both PBMC- and fibroblast-derived iPSC lines.

To overcome the expansion limits and donor dependence that constrain existing OC models, we sought to establish a conditionally expandable hematopoietic progenitor system that bypasses the lengthy and variable iPSC-to-OC differentiation route by enabling expansion and differentiation at a more advanced hematopoietic stage, thereby providing a faster and more reproducible human source for OC generation.

In this study, we utilized 5F (five transcription factors) hematopoietic stem and progenitor cells (HSPCs) to investigate their differentiation into OCs. 5F cells, or 5-factor cells, are lineage-respecified human HSPCs generated by introducing five transcription factors–HOXA9, ERG, RORA, SOX4, and MYB–into CD34^+^CD45^+^ myeloid progenitors derived from the human iPSC line MSC-iPS1. In the original approach by Doulatov et al. these iPSC-derived hematopoietic progenitor cells were transduced with doxycycline-inducible lentiviral vectors (pINDUCER-21 backbone) encoding the five factors. HOXA9, ERG, and RORA were sufficient to confer *in-vitro* self-renewal/multipotency, while the addition of SOX4 and MYB enabled reproducible short-term myelo-erythroid engraftment *in vivo*. Thus, 5F cells are best described as doxycycline-regulated, conditionally expandable, conditionally immortalized cells (Doulatov et al., 2013).

Building on this foundation, we investigated their capacity to generate functional, bone-resorbing OCs *in vitro*.

## Materials and methods

### 5F progenitor culture

5F progenitor cells were a kind gift from the Doulatov lab (University of Washington, Seattle, WA). They were cultured in StemSpan SFEM II medium (STEMCELL Technologies, Vancouver, Canada) to which 50 ng/mL human stem cell factor (hSCF), 50 ng/mL human thrombopoietin (TPO), 50 ng/mL human interleukin 6 (hIL-6), 10 ng/mL human interleukin 3 (IL-3), 50 ng/mL human Flt-3 ligand (Flt3L) (Peprotech, Cranbury, NJ, USA) and 2 μg/mL doxycycline (Sigma-Aldrich, St. Louis, MO, USA) were added.

### Osteoclast differentiation of 5F cells

Three experimental groups were established: (1) untreated 5F cells as baseline control, (2) 5F cells treated with M-CSF alone as a treatment control, and (3) 5F cells treated with a combination of M-CSF and RANKL to induce OC differentiation.

5F HSPCs were driven towards monocyte differentiation by incubation in X-VIVO 15 (Lonza, Basel, Switzerland) supplemented with 2 mM Ultraglutamine (Gibco,USA), 55 μM 2-mercaptoethanol (Thermo Fisher Scientific, Waltham, USA), 25 ng/mL hIL-3 (STEMCELL Technologies, Vancouver, Canada) and 100 ng/mL hM-CSF (STEMCELL Technologies, Vancouver, Canada) for 7 days at 37 °C in a 5% CO_2_ incubator. Following monocyte differentiation, cells were seeded in 6-well plates at 2 x 10^5^ cells/cm^2^ in α-MEM (Lonza, Basel, Switzerland), supplemented with 10% heat-inactivated fetal bovine serum (FBS) and 50 ng/mL M-CSF, and cultured for 3 days. Thereafter, the medium was replaced every other day for 7 days with fresh medium containing 80 ng/mL human RANKL (STEMCELL Technologies, Vancouver, Canada) and 50 ng/mL hM-CSF. Terminal differentiation was induced by culturing cells for 2 days in medium containing only RANKL. All experimental groups were subsequently subjected to functional characterization to assess OC differentiation, multinucleation, and bone-resorbing activity.

### TRAP staining

In order to assess OC morphology and confirm expression of TRAP, OC differentiation was performed on 8-well chambered µ-slides (ibidi, Gräfelfing, Germany). After differentiation, cells were fixed with 4% paraformaldehyde (PFA) (Boster Biological Technology, Pleasanton, CA, USA) and TRAP staining was performed using a TRAP staining kit (Sigma-Aldrich, St. Louis, MO, USA) according to the manufacturer’s instructions. OCs were then counterstained with methyl green nuclear stain (Sigma-Aldrich, St. Louis, MO, USA) for 10 min at room temperature. Image acquisition was performed using an inverted microscope (Nikon Ti2, Tokyo, Japan), and images were analyzed for cell size and multinucleation using ImageJ.

### Immunofluorescence

Following OC differentiation, cells were fixed with 4% PFA, permeabilized with Triton X-100 (Thermo Fisher Scientific, Waltham, USA) for 30 min and blocked with normal goat serum for 60 min. Following permeabilization and blocking, OCs were stained with a primary Anti-Cathepsin K antibody (Abcam, Cambridge, UK), followed by an Alexa 647-conjugated secondary antibody (Abcam, Cambridge, UK). Cells were additionally stained with TRITC-conjugated phalloidin and DAPI to visualize actin filaments and nuclei, respectively. Images were acquired using a Leica SP8X confocal laser scanning microscope (CLSM) and analyzed using ImageJ.

### Bone resorption assay

To determine the resorptive capacity of differentiated OCs, monocyte-differentiated and M-CSF mature 5F cells were seeded onto 48-well calcium-phosphate (CaP) resorption assay plates (Cosmo Bio, Japan) and onto bovine cortical bone slices (boneslices.com, Denmark). After terminal OC differentiation, cells were removed using 5% bleach. To quantify the resorption area on CaP plates, tiled full-well images were acquired. In addition to phase-contrast mode, images were captured using the yellow channel in fluorescence mode to heighten the contrast between resorbed and unresorbed areas. Quantification was performed in ImageJ by applying a manual threshold to these fluorescent images to calculate the percentage of resorbed surface area. Bone slices were stained with 0.1% Toluidine blue to visualize resorption pits.

### Gene expression analysis

RNA extraction from 5F HSPCs and differentiated cells was performed using the RNeasy Micro Kit (Qiagen, Hilden, Germany) according to the manufacturer’s instructions. Briefly, cultured cells were washed once with 1× PBS, lysed with 350 µL lysis buffer, and mechanically disrupted by scraping. RNA was purified using spin-columns and eluted in nuclease-free water. The RNA concentration was quantified with a NanoDrop spectrophotometer (Thermo Fisher Scientific, Waltham, USA). A total of 500 ng RNA was reverse transcribed into complementary DNA (cDNA) using the Omniscript Reverse Transcription Kit (Qiagen, Hilden, Germany). Gene expression of NFATC1, CTSK, MMP9, CA2, CSF1R, and TNFRSF11A were analyzed, with β-actin serving as the endogenous control.

### Flow cytometry

To assess the differentiation of 5F cells, flow cytometry was performed at the initial hematopoietic progenitor stage, following monocyte differentiation and after OC differentiation. OC differentiation was carried out on 6-well plates. On day 7, the differentiation medium was removed, and cells were rinsed with PBS. Cells were then incubated with 300 µL Accutase for 40 min at 37 °C in a 5% CO_2_ incubator. Accutase was subsequently neutralized with medium, and cells were collected by centrifugation at 300 *g* for 5 min. Finally, cells were resuspended in pre-cooled FACS staining buffer for subsequent flow cytometry staining. Cells were first blocked for Fc receptors using TruStain FcX (BioLegend, San Diego, USA), followed by staining at 4 °C for 1 h in the dark with primary antibodies against CD34 (PE-Cy7-conjugated, BD Biosciences, Franklin Lakes, NJ, USA), CD45 (APC-conjugated, BD Biosciences, Franklin Lakes, NJ, USA), CD14 (BV711-conjugated, BD Biosciences, Franklin Lakes, NJ, USA), CD11b (BUV-496 conjugated, BD Biosciences, Franklin Lakes, NJ, USA) and CD265/RANK (PE-conjugated, Thermo Fisher Scientific, Waltham, USA).

After staining, cells were fixed with 4% PFA. Positive control and compensation controls were performed with UltraComp eBeads (Thermo Fisher Scientific, Waltham, USA). Data was acquired using a BD symphony A3 (BD, Franklin Lakes, USA) and analyzed with FlowJo.

### Statistical analysis

Statistical analyses were conducted using GraphPad Prism 10 (GraphPad Software, San Diego, CA, USA). Non-parametric statistical tests were utilized to evaluate significance across all experiments. The Mann-Whitney U test was applied for comparisons between two groups, and the Kruskal–Wallis test was used for multiple group comparisons, as specified in the individual figure legends. Where appropriate, one-tailed tests were applied for directional hypotheses, as indicated in the respective figure legends. A p-value of less than 0.05 was considered statistically significant (*p < 0.05, **p < 0.01, ***p < 0.001, ****p < 0.0001).

## Results

### Tartrate-resistant acid phosphatase activity

**FIGURE 1:**
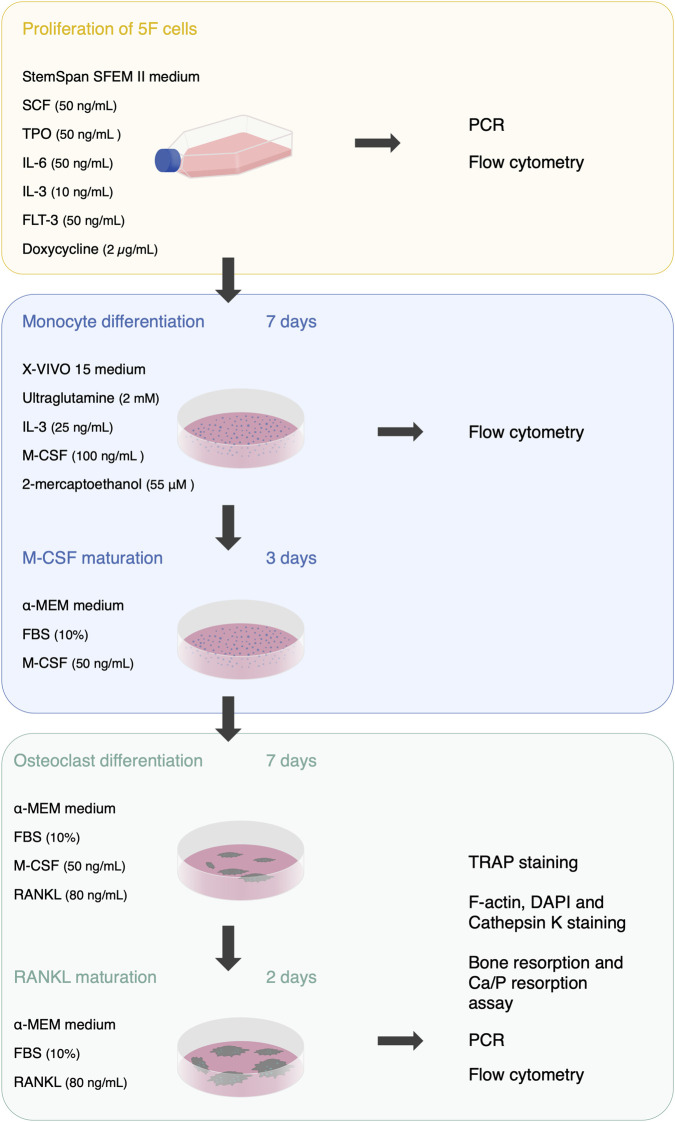
Schematic outline of the differentiation process.

A schematic summary of the protocol for 5F differentiation to OCs is outlined in Figure 1.

**FIGURE 2:**
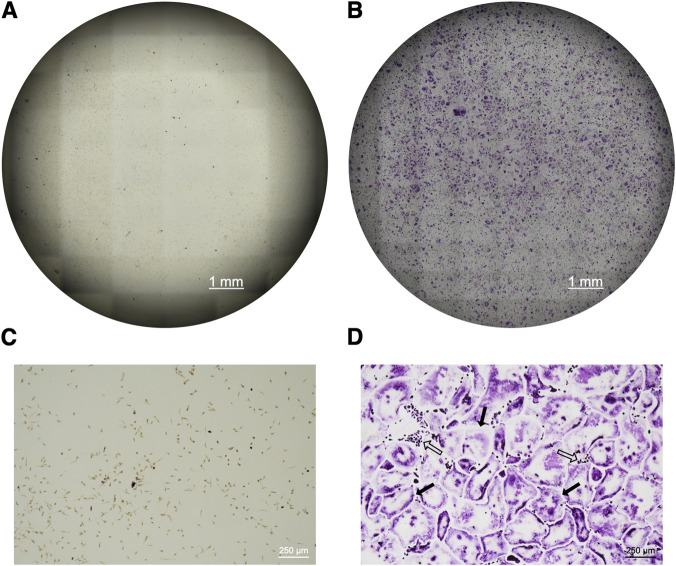
TRAP staining of cells after osteoclast differentiation, counterstained with methyl green. **(A)** Full-well image of the M-CSF control group showing no multinucleated, TRAP-positive cells, whereas **(B)** M-CSF + RANKL treatment induces numerous multinucleated, TRAP-positive osteoclasts. Higher-magnification views of **(C)** the control group confirm the absence of TRAP-positive osteoclasts, while M-CSF + RANKL-treated cultures display large, multinucleated, TRAP-positive cells characteristic of mature osteoclasts. Solid arrows indicate multinucleated OCs, while the open arrow highlights mononucleated cells. Scale bars: **(A,B)** = 1 mm; **(C,D)** = 250 µm.

Following monocyte differentiation, 5F cells were further differentiated into OCs as described below. The optimal monocyte seeding density for osteoclast differentiation was determined to be 2 × 10^5^ cells/cm^2^. Full-well images of the control group which were treated with 50 ng/mL human macrophage colony-stimulating factor (hM-CSF) (Figure 2A) showed no multinucleated cells. In contrast, treatment with 50 ng/mL hM-CSF and 80 ng/mL receptor activator of NF-κB ligand (RANKL) induced the formation of large, multinucleated TRAP-positive cells (Figure 2B). Upon higher magnification the control group (Figure 2C) did not exhibit multinucleated cells while higher magnification images the treatment group (Figure 2D) depicted large, multinucleated, tartrate-resistant acid phosphatase (TRAP)-positive OCs (pointed out by open arrows). Cells containing three or more nuclei were classified as OCs, with some containing more than 50 nuclei. On average, 203 OCs per cm^2^ (±8.2 SD) were observed.

### F-actin and cathepsin K staining

**FIGURE 3:**
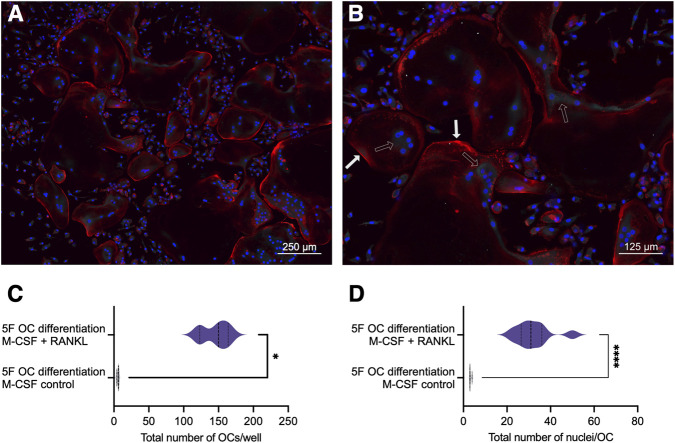
Morphological characterization of 5F-derived osteoclasts by confocal laser scanning microscopy (CLSM). **(A)** Large multinucleated osteoclasts outlined by prominent F-actin rings (solid arrows) containing up to 50 nuclei (blue) were observed. **(B)** Higher-magnification images show focal cathepsin K expression (turquoise, open arrows). **(C)** Quantification of osteoclasts per well demonstrated that 5F cells treated with M-CSF + RANKL generated significantly more osteoclasts than controls. **(D)** The number of nuclei per osteoclast ranged from 3 to 60. Scale bars: A = 250 μm; B = 125 µm. Statistical analysis was performed using the non-parametric Mann-Whitney U test (one-tailed for *n* = 3 biological replicates **(C)** and *n* = 6 replicates **(D)**, *p < 0.05, ****<0.0001).

OCs were further characterized by immunofluorescence staining for cathepsin K (turquoise) and F-actin (red), with DAPI as a nuclear counterstain (blue), followed by confocal laser scanning microscopy (CLSM) (Figure 3). 5F-derived OCs contained up to 50 nuclei per cell (Figure 3A). Higher-magnification images revealed prominent F-actin cytoskeletal rings (solid arrows) and focal cathepsin K expression (turquoise, open arrows) (Figure 3B). Quantification of OCs per well (Figure 3C) and nuclei per OC (Figure 3D) demonstrated significant increases in the RANKL-treated group compared with controls. A complete set of representative confocal images, including corresponding control conditions and comparative progenitor systems, is provided in Supplementary Figure S1. On average, RANKL-treated cultures contained 146 (±20.8 SD) OCs per well, each with approximately 32 nuclei (±8.2 SD).

### Bone resorption on calcium-phosphate substrate and bone slices

**FIGURE 4:**
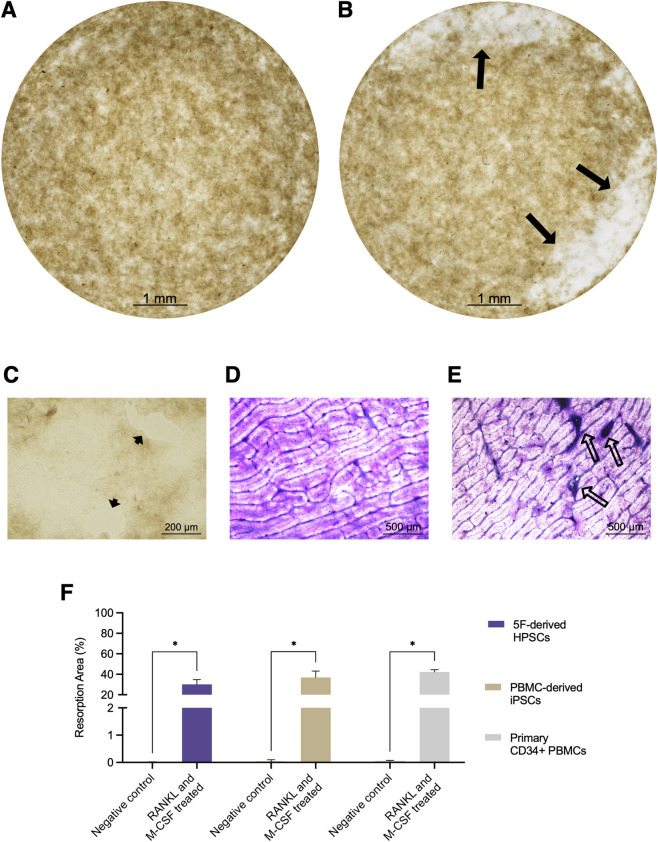
Bone resorption assay performed on calcium-phosphate-coated plates and bone slices. **(A)** Full-well image of a calcium-phosphate-coated plate seeded with 5F cells and treated with M-CSF (control). **(B)** Calcium-phosphate-coated plate seeded with 5F cells and treated with M-CSF + RANKL, showing resorbed areas (solid arrows). **(C)** Higher magnification view of resorption pits (arrowheads). **(D)** Bone slices seeded with M-CSF-treated 5F cells. **(E)** Bone slices seeded with M-CSF + RANKL-treated 5F cells, showing toluidine blue–stained resorption pits (open arrows). **(F)** Quantification of calcium phosphate resorption demonstrating robust osteoclast activity in 5F-derived cultures following M-CSF + RANKL treatment, with comparable resorptive activity observed in iPSC- and CD34-derived osteoclasts relative to negative controls. Scale bars: A, B = 1 mm; C = 200 μm; E, F = 500 µm. Statistical analysis was performed using the non-parametric Mann-Whitney U test (one-tailed for *n* = 3 biological replicates, *p < 0.05).

The resorptive activity of differentiated OCs was assessed using a calcium phosphate resorption assay and bone slices. No resorption was observed in control wells (Figure 4A), whereas M-CSF + RANKL–treated cells exhibited areas of resorption (Figure 4B, solid arrows) and distinct resorption pits (Figure 4C), including areas of complete calcium phosphate degradation that exposed the underlying plate surface (arrowheads). Regions of partial resorption were also evident when full-well images were compared with controls. Consistently, bone slices stained with 1% toluidine blue in sodium borate further validated osteoclastic activity as no resorption pits were observed in the control group (Figure 4D), whereas clear toluidine blue-stained resorption pits were detected in the RANKL-treated group (Figure 4E, open arrows). Quantitative analysis confirmed that 5F-derived OCs exhibited significantly greater resorptive activity than controls (Figure 4F), with an average resorption area of 33% in RANKL-induced cultures. Using OCs derived from iPSC progenitors and primary CD34^+^ hematopoietic progenitors as benchmark comparison systems, the resorption activity of 5F-derived OCs was comparable to that observed in iPSC-derived OCs and OCs differentiated from primary CD34^+^ progenitors.

### Osteoclast marker gene expression

**FIGURE 5:**
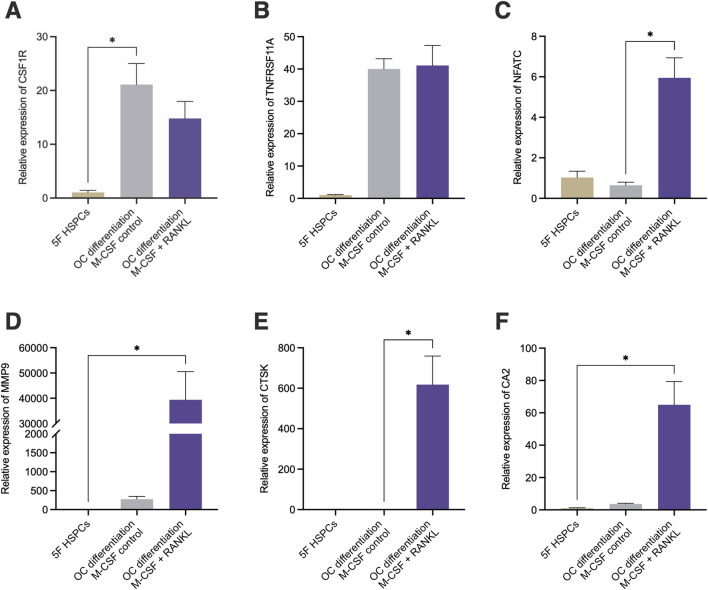
Gene expression analysis of osteoclast-related markers in 5F cells by quantitative real-time PCR (qRT-PCR). Expression was evaluated in untreated 5F cells, M-CSF controls, and M-CSF + RANKL–treated groups. **(A,B)** CSF1R and TNFRSF11A were weakly expressed in untreated cells but upregulated in both M-CSF and M-CSF + RANKL groups. **(C)** NFATC1, the master transcriptional regulator of osteoclastogenesis, was strongly induced by RANKL stimulation. **(D–F)** Osteoclast-specific markers MMP9, CTSK, and CA2 were significantly upregulated in the M-CSF + RANKL group. Statistics are based on non-parametric Kruskal–Wallis test (*n* = 3 biological replicates, *p < 0.05).

To determine whether 5F-derived OCs expressed OC-specific markers, gene expression was analyzed by quantitative reverse transcription (qRT)-PCR (Figure 5). Untreated 5F progenitors showed low expression of Colony-stimulating factor 1 receptor (*CSF1R*) (Figure 5A) and tumor necrosis factor receptor superfamily member 11A (*TNFRSF11A*, encoding RANK) (Figure 5B), both of which were upregulated during differentiation, with higher expression observed in the M-CSF control and M-CSF plus RANKL treatment groups. Nuclear factor of activated T cells 1 (*NFATC1*), the master transcriptional regulator of osteoclastogenesis, was significantly induced by RANKL stimulation (Figure 5C). Expression of matrix metallopeptidase 9 (*MMP9*) (Figure 5D), cathepsin K (*CTSK*) (Figure 5E), and carbonic anhydrase 2 (*CA2*) (Figure 5F) was weak in untreated progenitors and in the M-CSF control, but all three markers were significantly upregulated in the RANKL-treated group.

### Immunophenotypic characterization

**FIGURE 6:**
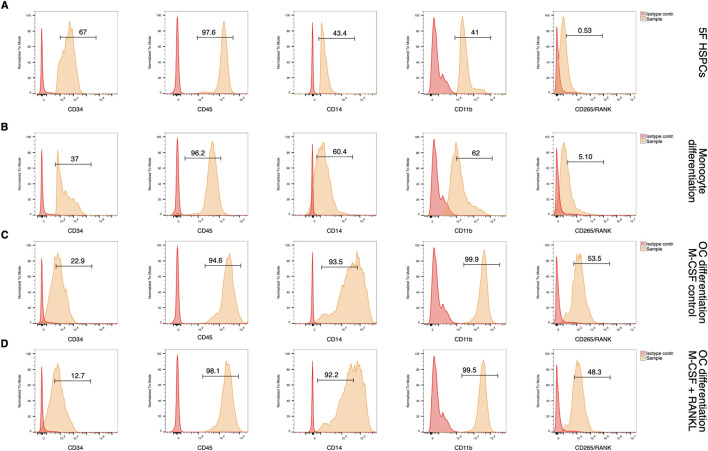
Flow cytometric analysis of surface marker expression during differentiation. Flow cytometry was performed on **(A)** 5F progenitor cells, after **(B)** monocyte differentiation and following treatment with **(C)** M-CSF alone or **(D)** M-CSF + RANKL. CD34^+^ cells (67%) progressively decreased, while CD45^+^ cells remained stable. CD14^+^ cells increased from 43.4% to 60.4% after myeloid differentiation and reached 93% after M-CSF + RANKL treatment. CD11b^+^ cells rose from 41% to 99%, and RANK/CD265^+^ cells increased from 0.53% to 50% across differentiation.

To evaluate hematopoietic and monocyte marker expression during differentiation, flow cytometry was performed on 5F HSPCs at the initial hematopoietic stage (Figure 6A), after monocyte differentiation (Figure 6B), and following OC differentiation with either M-CSF alone (Figure 6C) or M-CSF and RANKL (Figure 6D). At baseline, 67% of 5F cells expressed CD34, consistent with early hematopoietic progenitors (Figure 6A). This population progressively decreased during differentiation (Figures 6B–D). A large CD45^+^ population was present throughout and showed no major changes. By contrast, CD14^+^ cells increased from 43.4% at the hematopoietic stage to 60.4% after monocyte differentiation and further to 92% after M-CSF and RANKL treatment. Similarly, the CD11b^+^ population expanded from 41% to 99%. Notably, only 0.53% of 5F cells were initially RANK/CD265^+^, but this rose to 48% after OC differentiation.

## Discussion

In this study, we demonstrated that 5F HSPCs can be differentiated into functional, bone-resorbing OCs in the presence of M-CSF and RANKL. 5F cells were generated by transfecting iPSC-derived hematopoietic progenitor cells (HPCs) through the introduction of defined transcription factors, to respecify them toward a stem cell–like fate. Doulatov et al. showed that HOXA9, ERG, and RORA converted myeloid precursors into multilineage HSPCs with erythroid and lymphoid potential, while SOX4 and MYB enhanced myelo-erythroid engraftment. Building on this transcription factor–mediated reprogramming, we sought to determine whether 5F cells could provide a robust and scalable source of human OCs. Prior to OC differentiation, 5F HPSCs were directed toward the monocyte lineage following the protocol of Rössler et al., using IL-3 and M-CSF together with ultraglutamine to support metabolic activity and β-mercaptoethanol to maintain a reducing environment favorable for cell survival and proliferation (Rössler et al., 2020). This maturation step promoted the acquisition of monocyte-specific markers and primes the cells for subsequent RANKL-driven osteoclastogenesis.

Flow cytometry was performed on day 6 of OC differentiation, as beyond this point the cells adhere too firmly to the culture surface, become very large, and are highly susceptible to mechanical and shear stress, making them difficult to detach even with Accutase. As expected, CD34 expression decreased while RANK expression progressively increased during differentiation. CD14 expression was already detectable in untreated 5F cells. CD14 is a glycolipid-anchored membrane glycoprotein expressed in monocytes and macrophages (Schütt et al., 1999). It is also present on various hematopoietic and parenchymal cells and plays critical roles in cell differentiation, immune modulation, and host–pathogen interactions (Itoh et al., 2003). Importantly, CD14 has been reported as a marker of OC precursors (Blair and Zaidi, 2006; Tokunaga et al., 2020). Consistent with this, we observed a progressive increase in CD14 expression during differentiation. Since cells were collected on day 6, prior to full maturation, the elevated CD14 levels likely represent an intermediate stage of OC development.

Similarly, CD11b, which encodes ITGAM, non-covalently associates with the β-chain CD18 to form the integrin heterodimer Mac-1 (Khan et al., 2018). Hayashi et al. demonstrated that Mac-1 is essential for osteoclastogenesis, functioning as an adhesion molecule required for differentiation, and that its expression is upregulated by RANKL stimulation via NF-κB signaling (Hayashi et al., 2008). Yang et al. further showed that CD11b promotes RANKL-dependent OC differentiation through activation of the spleen tyrosine kinase (Syk) pathway (Yang et al., 2017). In agreement with these findings, our study revealed a progressive increase in CD11b expression throughout differentiation, supporting its role as a marker of early to intermediate OC development. Other integrins have also been reported to act early in osteoclastogenesis and to regulate adhesion, protrusive structures, precursor fusion and resorptive competence (Brockhaus et al., 2024). Beyond adhesion, CD11b/Mac-1 also contributes to precursor fusion and cytoskeletal organization, suggesting that its upregulation in our model reflects not only the differentiation state but also functional maturation of OCs.

NFATC1 is the master transcription factor regulating key OC-specific genes such as cathepsin K and TRAP, in cooperation with c-Fos (Kim et al., 2005; Winslow et al., 2006; Kim and Kim, 2014). In OC precursors, RANKL stimulation strongly induces NFATC1, which is initially localized in the cytosol and subsequently translocates to the nucleus following calcium-dependent phosphorylation, leading to auto-amplification (Asagiri et al., 2005). Omata et al. reported that NFATC1 expression is RANKL-dependent, with levels peaking on day 2 after stimulation (Omata et al., 2023). Christensen et al. further showed that matrix metalloproteinase-9 (MMP9) is cleaved and activated by cathepsin K (CTSK). Together, MMP9, an endopeptidase involved in extracellular matrix remodeling, and CTSK play central roles in bone resorption and have also been implicated in pathological processes such as breast cancer progression and bone metastasis (Christensen and Shastri, 2015). Consistent with these findings, we observed significant upregulation of NFATC1 in 5F-derived progenitors treated with M-CSF and RANKL, confirming activation of canonical osteoclastogenic signaling in this model. Importantly, this increase in NFATC1 was accompanied by upregulation of downstream OC markers, including MMP9 and CTSK, further supporting successful differentiation toward functional OCs.

Several studies have established protocols for OC differentiation from various sources, including PBMCs, bone marrow, cord blood cells, CD14^+^ monocytes, and the RAW264.7 cell line (Gerberding and Yoder, 1993; Eeles et al., 2015; Kalantari et al., 2016; Riedlova et al., 2023). Amano et al. also established the 4B12 cell line from mouse embryonic calvaria, which can be differentiated into OCs with M-CSF and RANKL (Amano et al., 2009). The number of multinucleated cells and the extent of bone resorption vary depending on the source, with CD14^+^ monocyte-derived OCs showing the highest resorptive activity (Riedlova et al., 2023). Likewise, when hematopoietic progenitors are myeloid-restricted, they generate robust OC populations with strong resorptive function (Blümke et al., 2023). In this context, our study demonstrates that 5F HSPCs provide a reliable alternative source, capable of producing large numbers of functional OCs with high resorptive activity. Furthermore, 5F HSPCs can be maintained in culture for several weeks in the presence of doxycycline while retaining proliferative potential, offering a scalable and reproducible system for long-term OC research and disease modeling (Doulatov et al., 2013).

A limitation of this study is the lack of distinction between OCs and other multinucleated giant cells such as foreign body giant cells (FBGCs) or Langerhans-type cells (Cai et al., 2024). Among these, FBGCs are most closely related to OCs, as they share several morphological features, including podosomes, F-actin rings, and TRAP activity, and are therefore frequently studied in parallel. FBGCs arise from the fusion of macrophages in response to foreign material and, unlike OCs, lack ruffled borders and show only limited expression of cathepsin K (Khan et al., 2013; Harkel et al., 2015). This distinction is relevant to our findings, as nearly 80% of cells were TRAP-positive, yet bone resorption activity appeared lower than in previous experiments (Blümke et al., 2023). It is therefore possible that a minor subset of the multinucleated cells represented FBGCs rather than true OCs. Doulatov et al. previously noted that 5F HSPCs states may be incompatible with *in vivo* applications, as reprogramming with the reported factors has not yet achieved permanent maintenance, regeneration, or full multilineage potential (Doulatov et al., 2013). Nonetheless, this study is the first to demonstrate that 5F cells are capable of undergoing osteoclastogenesis. Importantly, transduction with the five defined transcription factors does not appear to interfere with OC differentiation.

Compared to conventional OC models, 5F cells offer several advantages. As they are derived from human iPSCs, they more closely resemble human OC biology than murine cell lines such as RAW264.7.

In the conventional approach, scale-up of OC production is achieved during the iPSC propagation phase, as iPSC-derived HPCs have very limited capacity for expansion. Unlike iPSC-based approaches, which require scale-up prior to mesoderm and hematopoietic specification, 5F HSPCs can be conditionally propagated and expanded at a more downstream hematopoietic stage, enabling larger and more reproducible yields. A comparative overview of proliferation and differentiation trajectories across 5F-derived, iPSC-derived, and primary CD34^+^ progenitor systems is provided in Supplementary Figure S2, illustrating that while both iPSCs and 5F cells can be expanded, iPSC-based workflows require substantially longer culture periods due to the necessity to undergo mesodermal and hematopoietic differentiation prior to osteoclastogenesis.

In practical terms, conventional human OC models predominantly rely on donor-derived monocytes, hematopoietic progenitors from bone marrow or cord blood, or iPSC-based hematopoietic systems. While these approaches can yield physiologically relevant OCs, they are often constrained by limited material availability, high donor variability, and complex differentiation protocols. iPSC-based models, although renewable, depend on expansion at the pluripotent stage and require multistep mesodermal and hematopoietic differentiation, leading to long culture times and heterogeneous outcomes. In contrast, the 5F platform enables conditional expansion at a defined downstream hematopoietic stage, bypassing the variable early iPSC phase and allowing direct cytokine-induced differentiation into OCs. This downstream expansion strategy allows cell amplification at a defined hematopoietic stage, thereby reducing variability associated with early iPSC differentiation and shortening the time to functional osteoclast formation. The resulting 5F-derived OCs are large, multinucleated, and strongly resorptive, reflecting key features of primary human osteoclasts. Collectively, these characteristics position the 5F system as a renewable, efficient, and standardized platform for osteoclast biology, bone remodeling, and translational research.

## Conclusion

In conclusion, 5F cells represent a promising alternative source for generating OCs. Their differentiation into functional OCs is straightforward and provides a reproducible and scalable source of myeloid-derived cells, reinforcing their utility as a model for OC biology and related disease research.

## Data Availability

The raw data supporting the conclusions of this article will be made available by the authors, without undue reservation.

## Glossary

ANOVA: Analysis of variance
BSA: Bovine serum albumin
CA2: Carbonic anhydrase 2
CD: Cluster of differentiation
cDNA: Complementary DNA
CLSM: Confocal laser scanning microscopy
CSF1R: Colony stimulating factor 1 receptor
CTSK: Cathepsin K
DAPI: 4′,6-diamidino-2-phenylindole
EB: Embryoid body-based
FBGCs: Foreign body giant cells
FBS: Fetal bovine serum
Flt3L: FMS-like tyrosine kinase 3 ligand
HPCs: Hematopoietic progenitor cells
HSPCs: Hematopoietic stem and progenitor cells
IL-3: Interleukin 3
iPSC: Induced pluripotent stem cells
M-CSF: Macrophage colony-stimulating factor
MEM: Minimum essential medium
MMP9: Matrix metallopeptidase 9
NFATC1: Nuclear factor of activated T cells 1
OC/OCs: Osteoclast/Osteoclasts
PBMCs: Peripheral blood mononuclear cell
PBS: Phosphate buffered saline
PFA: Paraformaldehyde
qRT-PCR: Quantitative reverse transcription polymerase chain reaction
RANK: Receptor activator of nuclear κB
RANKL: Receptor activator of nuclear κB ligand
SCF: Stem cell factor
TPO: Thrombopoietin
TRAP: Tartrate-resistant acid phosphatase
