# Supplementary figures and images for: From 5F hematopoietic progenitors to osteoclasts: a scalable human model of osteoclastogenesis
Source: Front Cell Dev Biol. 2026 Jun 8;14:1773507. doi: 10.3389/fcell.2026.1773507 (PMC13284159; doi:10.3389/fcell.2026.1773507)

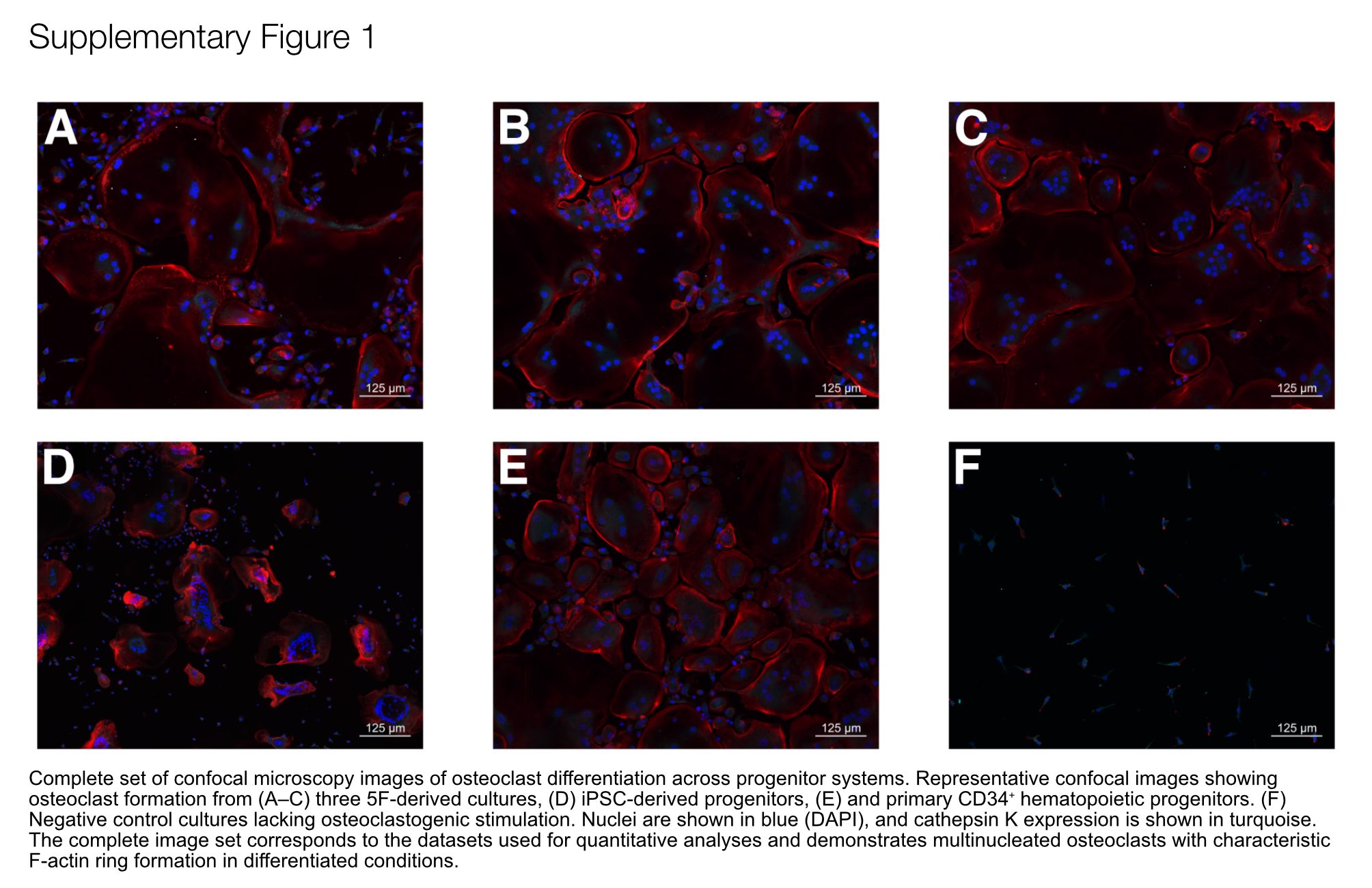

Supplement: Supplementary file 1 [file Image1.tiff]

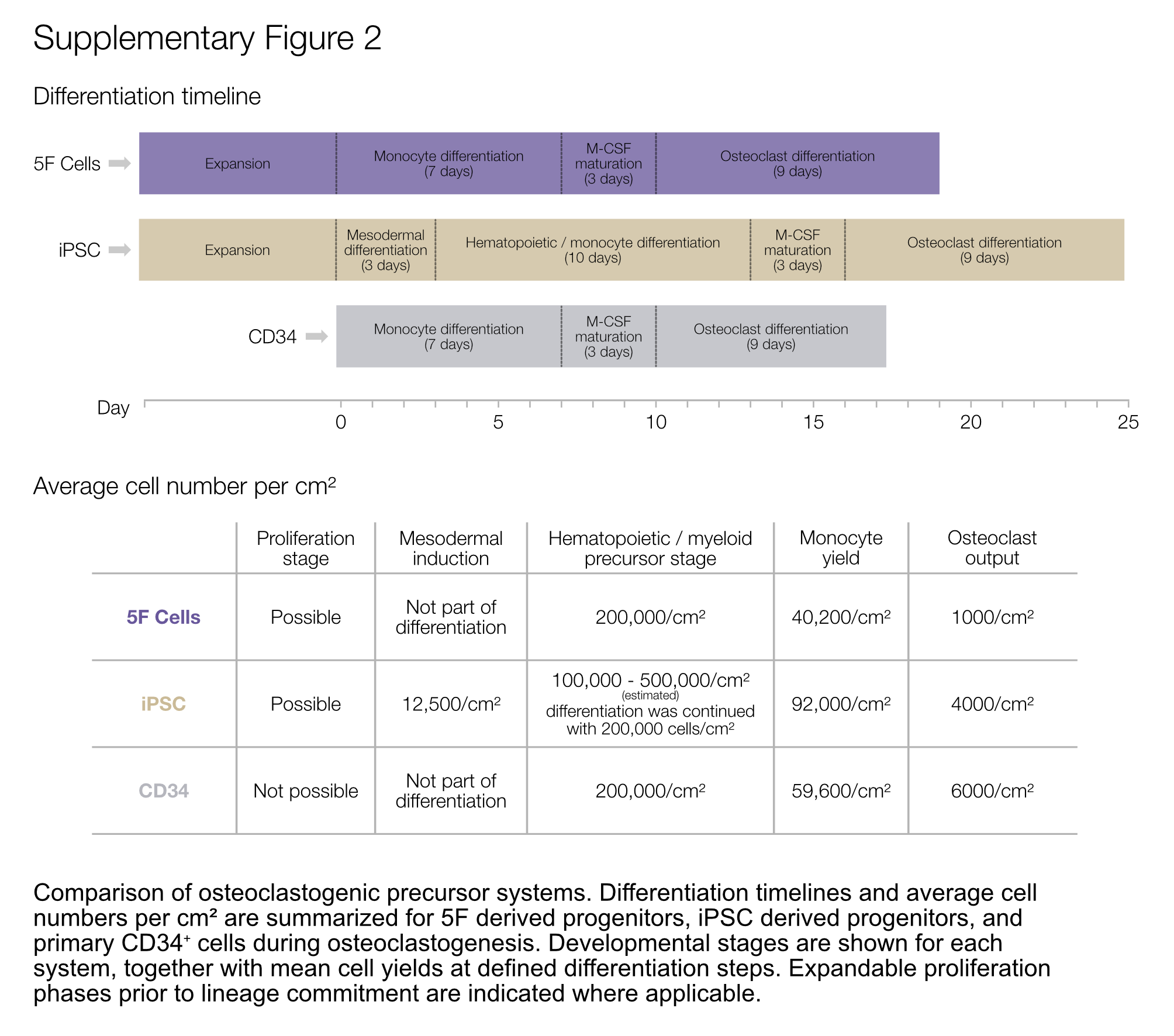

Supplement: Supplementary file 2 [file Image2.tiff]
